# Rational design of HIV vaccine and microbicides: report of the EUROPRISE annual conference

**DOI:** 10.1186/1479-5876-8-72

**Published:** 2010-07-26

**Authors:** Britta Wahren, Priscilla Biswas, Marie Borggren, Adam Coleman, Kelly Da Costa, Winni De Haes, Tessa Dieltjens, Stefania Dispinseri, Katrijn Grupping, David Hallengärd, Julia Hornig, Katja Klein, Lara Mainetti, Paolo Palma, Marc Reudelsterz, Janna Seifried, Philippe Selhorst, Annette Sköld, Marit J van Gils, Caroline Weber, Robin Shattock, Gabriella Scarlatti

**Affiliations:** 1 Karolinska Institute, Stockholm, Sweden; 2 San Raffaele Scientific Institute, Milan, Italy; 3 Lund University, Lund, Sweden; 4 Imperial College, London, UK; 5 St. George University, London, UK; 6 Institute of Tropical Medicine, Antwerp, Belgium; 7 Università degli Studi di Milano, Milan, Italy; 8 Università Vita-Salute San Raffaele, Milan, Italy; 9 University of Rome "Tor Vergata", Ospedale Pediatrico Bambino Gesù, Rome, Italy; 10 Robert Koch Institute, Berlin, Germany; 11 Institut de Biologie et Chimie des Protéines, Lyon, France; 12 Academic Medical Center, Amsterdam, the Netherlands

## Abstract

EUROPRISE is a Network of Excellence sponsored from 2007 to 2011 by the European Commission within the 6th Framework Program. The Network encompasses a wide portfolio of activities ranging from an integrated research program in the field of HIV vaccines and microbicides to training, dissemination and advocacy. The research program covers the whole pipeline of vaccine and microbicide development from discovery to early clinical trials. The Network is composed of 58 partners representing more than 65 institutions from 13 European countries; it also includes three major pharmaceutical companies (GlaxoSmithKline, Novartis and Sanofi-Pasteur) involved in HIV microbicide and vaccine research. The Network displays a dedicated and informative web page: http://www.europrise.org. Finally, a distinguishing trait of EUROPRISE is its PhD School of students from across Europe, a unique example in the world of science aimed at spreading excellence through training.

EUROPRISE held its second annual conference in Budapest in November, 2009. The conference had 143 participants and their presentations covered aspects of vaccine and microbicide research, development and discovery. Since training is a major task of the Network, the students of the EUROPRISE PhD program summarized certain presentations and their view of the conference in this paper.

## Introduction

Budapest, Hungary, hosted the second annual conference of the EUROPRISE Network of Excellence (NoE) from the 15th to the 18th of November 2009. The Network has organized several conferences, workshops and PhD courses on specific topics related to HIV vaccines and microbicides. To facilitate access to information, it provides a weekly newsletter edited by Anne-Marie Prieels from GlaxoSmithKline BIO that is freely accessible on the web homepage http://www.europrise.org. This is one of the first e-newsletters about HIV and the first to simultaneously cover the broad fields of prevention, science and technology, as well as policy aspects. It covers most scientific publications on HIV research and the most relevant news from the media.

The PhD School has 20 students directly receiving stipends from EUROPRISE and about 30 additional students, who, through their supervisors or collaborations, attend courses and meetings given by the network. The EUROPRISE training program has enhanced the students' possibilities to get involved in new collaborations with other scientific groups in Europe. This provides invaluable opportunities for students to prepare and deliver their scientific work in the form of abstracts, posters and oral presentations at meetings, including this annual conference.

The complete conference program is available at the EUROPRISE website http://www.europrise.org. Overview lectures concentrated on microbicide use, HIV vaccine design and trials in developed and developing countries. The lectures addressed the biological and medical aspects of vaccine and microbicide research, which are fundamental for basic research.

This article presents the students' own selection of presentations and is not meant to be a comprehensive coverage of the EUROPRISE second annual conference.

## Harmonization

An important issue for this large network is the standardization and reproducibility of assays to facilitate cross comparison and validation of data produced by the partners.

### Measuring immune responses

One workpackage of the network is devoted to harmonize assays for the measurement of cytokine secretion as a marker of cellular immune responses [1]. Richard Stebbings from NIBSC, Potters Bar presented a new standard for ELISpot and intracellular cytokine staining (ICS) assays. For this purpose, peripheral blood mononuclear cells (PBMC) were stimulated in the presence of a secretion inhibitor to accumulate intracellular cytokines. The cells were stabilized and suspended in freeze-drying buffer for long term stability to be used as lyophilized stimulated cell standards. Once reconstituted, these cells can be enumerated with cytokine based assays. Within the network, a collaborative study was performed to evaluate the lyophilized stimulated cell standards using both ICS and ELISpot assays. For comparison of results, partners received standard reagents for intracellular staining by FACS (detecting IL-2, IFNγ and TNFα) and for ELISpot assays (detecting IFNγ and TNFα) together with a strong and a weak cell positive control and the corresponding negative cell controls. All the participants placed the negative, weak and strong positive controls in the correct order, albeit with differing levels of sensitivity. Overall, less variability was found in ELISpot than in the ICS assay results. Taken together, these preliminary results demonstrate that lyophilized stimulated cells represent a good standard for the harmonization of cellular cytokine-based assays. Nevertheless, there are still some qualitative issues to be addressed, for instance cell size, debris and staining intensity of antigens. It also appeared that a longer stimulation and cytokine accumulation step would be needed to optimize ELISpot controls.

### Measuring neutralisation activity

Another workpackage of the network aims to develop, standardize and compare relevant assays for the detection of antibody responses. A previously initiated consortium, the NeutNet coordinated by Gabriella Scarlatti from San Raffaele Scientific Institute in Milan involves 18 independent laboratories from 12 EU countries. These participants showed in a first stage of activity that the sensitivity of different neutralisation assays differ, depending on both the antibodies and the virus used [2]. A second stage of NeutNet's work focused on comparing 8 polyclonal reagents against a panel of viruses in 17 different assays http://www.europrise.org/neutnet.html, utilizing uncloned virus supernatant (virus infectivity assays-VIA) or Env pseudotyped viruses (PSV assays) [3]. Target cells included PBMCs and engineered cell lines in single- or multiple-cycle infection formats. A comparison of findings showed a variation of neutralisation by a combination of three broadly neutralising monoclonal antibodies (TriMab) in both the PSV and the VIA assays. For the PBMC-based VIA, Inhibitory concentration (IC) 50 showed more variation than IC75 and IC90. In general, PSV assays were not more sensitive than VIA. Again, the variation was dependent on both the sera and the viruses that were used. Specific assay-to-assay comparison showed an important impact also of the target cell used. As of now, protective HIV neutralising immunity *in vivo *has not been defined. It is therefore recommended that more than one assay is used to obtain optimal information on the virus neutralisation potential of a serum or agent.

## Susceptibility to HIV-1 infection

Guido Poli from the San Raffaele Scientific Institute in Milan presented post-entry events of viral infection. The discovery of CCR5 and CXCR4 as obligatory entry co-receptors in CD4 + cells (T lymphocytes, macrophages and dendritic cells) has resulted in creation of novel antiretroviral agents targeting these host determinants. Some of these are also in development as potential microbicide candidates. This knowledge has also contributed to an understanding of the distribution of HIV-1 variants, both world-wide and inter-individually. In acute infection there is a disproportionate distribution of HIV-1 strains using exclusively CCR5 as entry coreceptors (R5 viruses), while CXCR4 utilization (usually in association with CCR5) mostly occurs in subtype B infection in late stages of disease [4]. HIV-1 transmission on the other hand most frequently occurs from R5 viruses, even when the transmitter harbours a CXCR4-using virus as the dominant quasi species. A central issue is the understanding of whether the asymmetric distribution of HIV-1 transmission can be explained by the efficiency of viral entry into the targets cell or by post-entry effects. Previous work [5] has demonstrated that CCR5-dependent R5 viruses replicate in primary CD4 + T-cells of cord blood origin, while CXCR4-dependent X4 viruses do not. This replication can be mimicked *in vitro *in interleukin-2 enriched medium after initial mitogenic stimulation.

Using a modified version of this protocol, Poli and collaborators have analysed a transcriptome of the host genome of about 22,000 genes, 11% of which were found to be activated by either R5 or X4 infection (productive and non-productive). The group is also optimizing a similar model system using peripheral CD4 + T-cells obtained from both healthy children and from children affected by primary immunodeficiency collected before and after gene therapy. The results confirm the original finding that a post-entry permissive signal for HIV replication is delivered by R5, but not by X4 viruses.

## Mucosal immune responses to HIV

### Dendritic cell populations

The role of dendritic cells (DCs) in the detection, spread and control of HIV-1 infection has been under investigation for quite some time. At the EUROPRISE conference, Dominique Kaiserlian from INSERM in Paris and Maryse Peressin (PhD student) from the University of Strasbourg emphasized the role of DCs in HIV-1 infection. Since DCs play a major role in the induction of an adaptive immune response within the mucosa [6], it is crucial to understand the functions and roles of the various DC populations, including Langerhans' cells (LCs), dermal DCs (dDCs), blood-derived myeloid DCs (mDCs) and plasmacytoid DCs (pDCs).

Kaiserlian drew attention to the *in vivo *ability of mucosal and skin DCs to induce CD8 + CTL response and tolerance within monostratified (gastrointestinal tract) and pluristratified (buccal mucosa and skin) epithelia. Indeed, the nasal, buccal and intestinal mucosal tissues provide ideal surfaces for vaccine delivery to induce mucosal immune responses. However, the efficacy by which a vaccine can be delivered to such surfaces is hampered by mucosal tolerance, particularly in the gastrointestinal tract [7]. Hence, there needs to be a balance between immunological tolerance and immunity. As shown by intradermal vaccination into the skin or buccal mucosa, DC recruitment via the CCR6/CCL20 pathway, rather than resident mDCs, was responsible for *in vivo *cross-priming of CD8 + CTL responses. In addition, testing of adjuvants shown to induce local secretion of CCL20 was followed by strong antigen specific CD8 cross-priming. This suggests that targeting of specific DC populations in conjunction with local secretion of specific substances can lead to the induction of CD8 T-cells. One such pathway was shown to be the up-regulation of CCL20 in the epithelium followed by DC recruitment via CCR6. By a series of experiments Kaiserlian and collaborators concluded that newly recruited DCs, rather than tissue resident LCs or dDCs, prime CD8 + CTLs. As the balance between immune response and tolerance is important in the design of an HIV vaccine, Kaiserlian's group investigated the mechanism of oral tolerance against antigen delivered intragastrically. They showed that antigen leakage from the gastrointestinal tract to the liver allowed antigen uptake and presentation by tolerogenic pDCs in the liver and mesenteric lymph nodes. pDCs are essential for oral tolerance in that they induce T-cell hypo-responsiveness. Bypassing antigen uptake by pDC might thus be a way to circumvent oral tolerance and generate an anti-infectious oral vaccine.

Since DCs are implicated in the sexual transmission of HIV-1 [8], Maryse Peressin has investigated inhibitory activities of HIV-1-specific antibodies in the context of HIV-1 infection and DCs/T cells co-localization. In her talk on HIV-1 infection and cell-to-cell transfer in primary DCs/T-lymphocytes coculture, she addressed the role that HIV-1 specific antibodies might play, for instance, after sexual transmission of HIV. *In vivo*, DCs such as LCs or interstitial DCs (iDCs), located in mucosal epithelium or sub-mucosal tissue respectively, are considered to be the first HIV targets [9]. Moreover, these DCs have been demonstrated to transfer HIV to permissive CD4 + T-lymphocytes *in vitro*. Using an *in vitro *model of LCs and iDCs Peressin was able to show that immunoglobulin G (IgG) can prevent HIV infection of LCs and iDCs by two mechanisms: first, neutralisation of the virus *via *the Fab fragment of the antibody; second, inhibition of virus and/or infected cells infection mediated by the action of the antibody Fc receptor. Peressin demonstrated that non-neutralising IgGs also inhibited HIV infection of the LCs/iDCs via an Fcγ receptor dependant mechanism. This mechanism may also involve effector cells binding to the antibody Fc receptor. HIV transfer from LCs/iDCs to CD4 + T lymphocytes can be efficiently inhibited by non-neutralising IgGs, highlighting the need for vaccines to induce mucosal neutralising as well as non-neutralising Igs in order to prevent the initial establishment of infection [10].

### Dendritic cell maturation

Annette Sköld from the Karolinska Institute in Stockholm presented her work on the effect of different Toll-Like Receptor (TLR) ligands on monocyte-derived DC (moDC) evaluated by upregulation of CD80 surface expression. moDC are known to express TLR1 to TLR6 and TLR8, possibly also TLR9. Sköld showed that the TLR3 agonist Poly(I:C) and the TLR4 agonist LPS, but not CpG DNA (a TLR9 agonist), can induce maturation of moDCs. TLR9 activation worked together with TLR4 but not with TLR3 activation. The TLR3 activation induced the production of cyto/chemokines IL-12, IL-4, TNF-α, MCP-1, MIP-1α and MIP-1β. Phosphorylation of IRF3 occurred after TLR3 activation, but not after TLR9 activation by CpG DNA. The fast timing of these events suggests that the inhibitory effect of TLR9 ligands must occur early in the transduction pathway. Finally, Sköld presented a CpG DNA molecule with a modified backbone which did not induce moDC maturation, and was only partially able to inhibit the TLR3 Poly(I:C)-induced maturation. This implies that the structure of the fine structure of the oligonucleotide CpG DNA is essential for its inhibitory function [11].

### Mucosal immunity

Human mucosa displays a surface area of more than 400 m2 and contains roughly 80% of all immune cells. Hence, research has been focused on the development and improvement of mucosal adjuvants and routes of vaccine delivery to elicit mucosal immune responses. In the context of HIV-1 infection, mucosal priming events in particular are essential to elicit memory cells at sites of pathogen entry. As underscored by Donata Medaglini from the University of Siena, T-cell priming results may be important as early markers of vaccine immunogenicity and immunological memory. However, the study of T-cell priming is hampered by the low precursor frequency of naïve T-cells. Using an adoptive transfer model of naïve antigen-specific transgenic T-cells in singenic recipient mice, Medaglini's group tested the ability of various mucosal TLR-dependent as well as -independent adjuvants (CpG, LTK63, CTB, and α-GalCer), or of different vaccine delivery systems (*Streptococcus gordonii *and Adenovirus), to prime local CD4 + and CD8 T-cells. Vaccine formulations were administered either nasally or vaginally and T helper and CTL proliferation, expression of activation and migration markers were analysed [12]. Intranasal immunisation with recombinant *S. gordonii *vaccine vector allowed efficient internalization of the vaccine by DCs, followed by DC maturation and activation. The intranasal immunisation induced primed CD4 + and CD8 + T-cells in the lymph nodes draining the respiratory, genital and intestinal tract. This response was maintained post immunisation. Using this type of vaccination strategy, Medaglini and her group were able to observe activation of proliferating T-cells as measured by the up-regulation of CD69. At the same time, a modulation of migration markers such as CCR7 and CD62L could be detected on proliferating CD4 + and CD8 + T-cells. A comparison of routes for vaccine administration showed differences between intranasal and intravaginal administration. Although good proliferative responses were found in both scenarios in lymph nodes draining the immunisation site at day 5 post vaccination, proliferation in distal sites was observed primarily following intranasal vaccination. Overall, it was concluded that the adoptive transfer model is a powerful tool for studying priming by mucosal adjuvants and delivery systems *in vivo*.

### Mucosal adjuvants

The talk on mucosal adjuvants for the genital tract by Ali Harandi from the University of Göteborg highlighted the significance of the development of adequate mucosal adjuvants and their efficient delivery to the genital tract. Although mucosal vaccines have been approved for use in humans, no mucosal adjuvants have been licensed so far. Much attention has been paid to the use of TLR ligands as adjuvants, though their efficiency and safety as mucosal adjuvants in the vaginal tissue has yet to be confirmed. Harandi's group examined the effectiveness of CpG oligodeoxynucleotides (ODN), which are TLR9 ligands, and α-GalCeramide as potential mucosal adjuvants in the murine female genital tract. He reported that mice given an immunogen (herpes simplex virus, HSV) together with CpG ODN were protected against HSV challenge [13]. Similarly, α-GalCer was able to confer 80% protection to a subsequent HSV challenge [14]. This suggests that both reagents could potentially also be used as adjuvants in the human vaginal tract. The myeloid differentiation primary-response gene 88 (MyD88), regarded as one of the key signaling adaptor proteins for TLRs, activates the transcription factor NF-κB. This signaling pathway is considered essential for a protective innate response. However, as highlighted by Harandi, the development of an antibody response to live HSV-2 at the murine vaginal mucosa was MyD88- independent, suggesting that MyD88 is not essential for inducing acquired protection and, in this case, a genital mucosal immune response. Given that CpG ODN and α-GalCer both showed the potential to enhance mucosal immunity, Harandi's group investigated the effect of these adjuvants on global gene expression using a microarray for the whole mouse genome. They grouped genes commonly induced by adjuvants into various categories, including cytokines and chemokines. A group of common genes including those for CCL9 and CXCL11 was identified with an expression pattern in the vagina that was linked to the mucosal adjuvant. Ways of directly targeting these genes or proteins were discussed as means to induce a controlled mucosal immune response.

Safety is critical in the improvement of adjuvants and vaccines. Hence, the degree of inflammation and IL-1 production in the presence of such adjuvants in mucosal tissue was analysed α-GalCer was described to induce low levels of inflammation, while CpG oligonucleotides up-regulated a larger number of genes in vaginal tissue and induced inflammation to a greater extent [15]. Since increased inflammatory reaction may enhance HIV infection, Harandi strongly emphasized the importance of carefully identifying and selecting mucosal adjuvants for vaginal application in humans.

One of the major challenges in the field of HIV-1 prevention is to elicit both systemic and mucosal protection. To address this question, a novel approach of mucosal immunisation was evaluated by Quentin Sattentau from the University of Oxford; he combined an Env-based experimental vaccine antigen (gp140CN54) with PRO2000, a candidate topical microbicide (Wegmann F, Krashias G, Luhn K, Laamanen K, Jeffs SA, Shattock RJ, Sattentau QJ: A mucosal vaccine strategy for enhanced mucosal HIV-1 antibody responses in an anti-inflammatory environment, Submitted). This vaccine - microbicide combination, tested in mice and rabbits, significantly increased the titres of Env specific mucosal IgA (mice) and systemic and mucosal IgG (rabbits) compared to immunisation with Env alone. Furthermore, rabbit vaginal IgG was able to neutralise the virus. Moreover, PRO2000 was shown to be a robust TLR4 antagonist which may create a mucosal anti-inflammatory environment by skewing the mucosal immune response towards a Th2-type and suppressing the production of inflammatory mediators. This anti-inflammatory environment in combination with the induction of locally produced neutralising antibodies may provide a primary barrier to mucosal HIV-1 infection.

## Adaptive immunity parameters

### T cell maturation

Nicolas Ruffin's PhD project at the Karolinska Institute, Stockholm, concerns the adaptive immune response. Increase of CD28-CD8 + T-lymphocytes is characteristic for ageing and for chronic inflammatory infections such as HIV-1 [16]. The increase is a consequence of massive cell division, due to continuous immune responses to persisting viral antigens. The CD28- T-cells are considered to be at a final differentiation stage of antigen-activated cells. They are reported to be resistant to apoptosis and once generated they will persist. This could explain the increasing proportion in HIV infected individuals [17,18]. Work presented by Ruffin has focused on apoptotic and proliferative abilities of the CD28 + and CD28- T-cell populations in HIV infected individuals. High levels of CD28- T-cells were found in HIV-infected individuals, but surprisingly the levels were equally high in treatment naïve and treated individuals, demonstrating that antiretroviral treatment cannot restore CD28- T-cells to normal levels. The accumulated CD28- T-cells show a senescent phenotype and in treatment naïve patients they have a propensity to apoptosis. This finding is in contrast with a previous study reporting that CD28- T-cells were resistant to apoptosis [17]. The enhanced apoptotic ability could be due to viral replication, since the level of apoptotic cells correlated with viral load. A difference between treatment naïve and treated patients was also found with regard to the proliferating ability of CD28- T-cells. Cells from treated patients instead showed a strong proliferating ability in response to TCR triggering. Thus, the work by Ruffin shows that viral replication alters the T-cell homeostasis and functionality of CD28- T-cells.

### Induction of improved antibody responses

Donato Zipeto from the University of Verona talked about broad-spectrum neutralising antibodies against HIV-1 elicited by fusion complexes and CD4-independent gp120/41s. Due to the extraordinarily high variability of the HIV envelope glycoprotein, it is essential to focus on conserved epitopes. Such conserved epitopes are exposed transiently during fusion of the gp41 transmembrane region with the target cell. Immunisation of mice or rabbits with fusion complex intermediates or CD4-independent gp120/41s demonstrated that such complexes are immunogenic and induced antibodies [19]. Indeed, monoclonal antibodies produced from immunised animals could neutralize viruses expressing the envelope glycoproteins from diverse HIV-1 isolates.

One project focused on the possible role that broadly-neutralising antibodies might have in limiting HIV disease progression. However, Zelda Euler (PhD student) from the Academic Medical Center at the University of Amsterdam found no correlation between cross-reactive HIV-1 specific neutralising activity in serum and the clinical course of HIV-1 infection [20]. This study took advantage of the large Amsterdam Cohort of HIV-infected patients established before the highly effective antiretroviral compounds were used. Sera from 82 members of the cohort collected 3 years after seroconversion were tested for levels of cross-reactive neutralising activity and correlated with the length of time the patients remained free of disease. Broadly neutralising antibodies were present in the sera of 23 of the patients but this finding did not appear to be beneficial. The rate of progression to AIDS was similar to that of patients with no such neutralizing activity. Even more surprising was the observation that the presence of cross-reactive antibodies was associated with a lower CD4 + T-cell count at viral set point. The results therefore indicate that although the HIV envelope glycoprotein is highly immunogenic during natural infection, even the development of broadly cross-reactive neutralising antibodies does not appear to benefit the host. It seems likely that the virus present in the patient at the time of sampling and thereafter represents a neutralisation escape mutant. HIV-specific cytotoxic cells and other factors than antibodies appear to play a role in the continued capacity of the patient to maintain a deterred viral set point and prolong the progression to AIDS in the so called elite controllers [20].

Presentations by the PhD students Tessa Dieltjens, Lara Mainetti, Marie Borggren, Evelien Bunnik and Marit van Gils concerned the evolution of HIV-1 virus in response to the humoral immune pressure. A range of viruses from patients at various stages of disease progression were studied for susceptibility to neutralisation, using autologous sera or monoclonal antibodies. All viruses were shown to escape from the neutralising antibody response mounted by the host [21-23]. Interestingly, viruses with escape mutations were still susceptible to neutralization with autologous sera obtained at later time points or with certain monoclonal antibodies. Discussions of the potential mechanisms of viral escape attributed it to changes in the number, length and charge of potential N-linked glycosylation sites. It has been suggested that over time HIV-1 has adapted to the pressure exerted by the human immune system. On a similar note, the long-term use of antiretroviral drugs is expected to be reflected in the world-wide appearance of drug-resistant HIV.

### Novel cross-priming strategies

An HIV-1 vaccine solely based on inducing cellular immunity appears to be insufficient to protect against HIV-1 infection (for example, the Merck vaccine study). A particular challenge for the induction of a neutralising antibody response is to improve immunogen capturing, processing and presentation by antigen presenting cells (APC). Hans Wolf, from the University of Regensburg, presented a new approach that uses a novel technique to reactivate virus-specific cytotoxic T-cells and T-helper cells by means of cross-presentation of soluble proteins mediated by urea adjuvants.

Priming and re-stimulation of CD8 + CTL requires the endogenous processing of proteins and the expression of relevant fragments within the context of the MHC-I molecule, a process usually resulting from *de novo *intracellular protein synthesis. Exogenous proteins, despite being internalised by macrophages, do not usually enter this pathway and vaccines based on inactivated viruses or purified proteins are generally poor inducers of the CTL response. However, certain types of antigen presenting cells are able to facilitate the induction of CTLs upon exposure to protein antigens via the process of cross-presentation. Novel modifications of proteins have been developed to take advantage of this phenomenon. It was shown that dissolution of proteins in high molar urea followed by pulsing of cells in low urea concentrations can overcome the barrier to endogenous processing. The urea treatment facilitated protein translocation into both the MHC-I and the MHC-II presentation pathways. Using the Epstein-Barr Virus (EBV) BZLF1 protein, Wolf's group showed that the urea-treated EBV protein (uBZLF1) undergoes temperature-dependent uptake by APC and also that different sub-populations and mononuclear cells from EBV-seropositive individuals pulsed with uBLZF1 were efficiently induced BLZF1-specific cytotoxicity and T-helper cells by means of cross-presentation [24]. Similar results were obtained when this technique was applied to two cytomegalovirus proteins. Finally, the *in vivo *priming of cytotoxic T-cells was demonstrated in mice using urea-treated HIV-1 p24 combined with CpG oligonucleotides. Overall, Wolf stressed that urea treatment of proteins successfully induces antigen-specific CTL, and that this technology may be considered as a new strategy to increase protein-specific CTLs. This innovation may have prominent implications for *in vivo *priming of HIV-1-specific CTLs in therapeutic vaccine studies.

## Early Clinical Studies

Margarita Bofill from Irsicaixa in Barcelona investigated the effects of the administration of growth hormone (GH) on immune reconstitution of HIV-infected adults. HIV-infection causes a severe down-regulation of virus-specific CD4 + and CD8 + T-cells that is not restored upon treatment with highly active antiretroviral therapy (HAART) [25]. Bofill and collaborators analysed whether treatment with GH and HAART could lead to expansion of the thymus and thus restore antigen specific immune responses. One of the earliest associations linking GH with the thymus was the observation that thymic atrophy in aging individuals correlated with lower GH-levels [26]. Several authors have subsequently reported that GH affects T-cell function by promoting thymic function and progenitor survival as well as improving peripheral T-cell functions. Both GH and IGF-1 have been shown to increase T-cell functions *in vitro*. This suggests a role for recombinant human GH as a possible immunomodulatory therapy, complimentary to the benefits of effective antiretroviral drug therapy, for HIV-1 infection [27]. Patients with HAART and complete viral suppression who failed to elicit a humoral response to Tetanus Toxoid, or to Hepatitis A or to Hepatitis B virus were selected for this study and randomized in 3 groups: one group receiving HAART + GH + vaccines; another group receiving HAART + GH but not vaccines; and a control group receiving HAART + vaccines but no GH. GH was given for 6 months at the dosage of 3 mg/kg aiming to enhance thymic output and restore specific responses to vaccine antigens. The GH administration resulted in an increase of thymus volume in nearly 50% of the treated patients. This increase correlated with increased CD4 + counts and number of T regulatory cells, but not with the level of IL-7. Overall, recall responses to Hepatitis A, Tetanus Toxoid and HIV (p24-gag) seem to be restored in the majority of patients treated with GH compared to the other groups. Despite the high toxicity related to GH treatment reported in the literature, minor adverse events were observed in this trial in the short-term follow-up. Viral load was maintained under 50 copies/ml in all patients and no difference in proviral DNA was reported. Although long-term toxicity related to GH treatment seems to preclude large scale application of this strategy [28], this study shows that selective molecules targeting thymic function may represent a therapeutic option, particularly in those patients who are severely immunocompromised.

## Vaccines

A special guest of the meeting was Jerome Kim (U.S. Military HIV Research Program), who presented the recently concluded Phase III Prime Boost HIV Vaccine Trial performed in Thailand [29]. The ALVAC-HIV and AIDSVAX B/E combination used for the prevention of HIV-1 infection in young Thai adults showed for the first time in the HIV/AIDS vaccine era a modest effect of 31% protection on the acquisition of HIV-1 infection. The vaccination had a more pronounced efficacy in the low and medium risk groups (40 - 47% reduction) than in the high risk population (3.7% reduction). There was no difference in either early viral load or post-infection CD4 + T-cell count between vaccine and placebo groups. This study raises some important issues, such as the part played by the CD8 T-cell response, the definition of the impact of risk and the need to understand which arm of the immune response is working. Consideration should be paid to further efficacy trials, possibly based on this vaccine principle, in high risk cohorts.

### Model studies in macaques

Vaccination of Rhesus macaques with live attenuated SIV provides immediate protection against wild-type virus. While the use of attenuated HIV in humans is unlikely, the model may provide important insights on the mechanisms of protection. In order to investigate the role of adaptive immune responses and to understand whether the persistence of the vaccine virus is central to protection in macaques, a conditional live attenuated (Δnef) SIV has been developed that is dependent on the presence of doxycycline (SIVrtTA) [30]. SIVrtTA has proven to be infectious *in vivo*, with peak viremia slightly lower and kinetics similar to SIVmac 239Δnef [31]. The virus persisted in 2 out of 4 animals after doxycycline delivery had been terminated. Partial protection against challenge with wild-type SIVmac 239 was observed in vaccinated animals, but only in those with detectable SIVrtTA titers after removal of doxycycline. Thus, it was proposed that the persistence of the vaccine virus is crucial for protection; the hypothesis will be further investigated with the SIVrtTA model.

It is difficult to cross-calibrate the human and macaque models for immunisation studies; it is therefore crucial to design challenge experiments that resemble the human system as closely as possible. Hence, to reflect human transmission routes such as sexual intercourse, repeated low-dose mucosal challenges in monkeys may be performed instead of intravenous challenges [32]. HIV dynamics are fast, the time to act is short. The best time to immunise with a mucosal vaccine is the mid-point of the female follicular cycle, but this is easily missed. Constant immunisation, using a microbicide that causes constant effector function and simultaneously immune-stimulates mucosal surfaces, would bypass this problem. Cranage from St George's University of London showed that repeated intravaginal administration of HIV-1gp140 in macaques augments systemic and mucosal antibody responses following systemic priming with adjuvanted protein [33].

### Lentiviral vectors

Andrea Cara from the National AIDS Center in Rome summarised his work aimed at increasing the safety of lentiviral vectors. The use of such vectors for vaccination is currently cautious because of the potential dangers posed by integration of vector nucleic acids into the host genome. Integrase-defective lentiviral vectors (IDLV) based on HIV or SIV with mutational inactivation of the catalytic sites do not integrate. Transduction of both dividing and non-dividing cells results in transcription of episomal forms of the vector and a strong expression of the gene of interest. In non-dividing cells, episomal vector expression continues over a long period of time, whereas in dividing cells the expression decreases as the cells proliferate [34]. A single immunisation with gp120- expressing IDLV in mice resulted in vigorous immune responses, i.e. the induction of polyfunctional CD8 + T-cells and specific serum antibodies directed to gp120. Experiments using human DCs and macrophages transduced *ex vivo *with influenza M1-expressing vectors demonstrated that these cells induced a strong expansion of autologous, antigen-specific CD8 + T-cells. This suggests that replication-defective vectors could be used for safe and efficient transduction of human antigen-presenting cells for vaccination purposes.

### Vehicles and virus-like particles

Caroline Weber (PhD student) presented her work on the use of nanoparticles as a vaccine vehicle for the delivery of adjuvants and antigens. For this purpose, biodegradable synthetic Poly D, L-lactic acid (PLA) or chitosan (CNP) nanoparticles, which are readily phagocytosed by DCs, were used [35]. Phagocytosis of nanoparticles with immunomodulators, such as HIV-1 antigens, adsorbed onto them leads to the maturation of DC increasing MHC-I and II expression and other activation markers and the release of cytokines [35,36]. Weber demonstrated that the nanoparticles can be used to deliver HIV-1 antigens p24 and gp140 and that they could also be used to deliver TLR agonists to endosomal TLR3 and TLR7/8. Combining the delivery of vaccines and adjuvants using nanoparticles could improve conditions for potent antigen presentation. TLR agonists adsorbed to nanoparticles provide a synergistic effect in the maturation of DC, while agonists associated with other types of particles only showed additive effects. This type of particles are promising as potential vaccine or adjuvant delivery systems.

Luigi Buonaguro from the National Cancer Institute in Naples emphasized the advantage of virus-like particles (VLPs) in HIV-1 vaccine development. The concept of using VLPs to induce cellular immune responses has already been used in many areas of virus vaccine research, the reasons being their ability to structurally mimic the actual pathogenic agent and their potential to express multiple epitopes in order to induce a broad immune response. Buonaguro's group engineered VLPs expressing HIV-1 gp120 and Pr55gag and used them for intraperitoneal and intranasal immunisation of mice [37]. Intranasal vaccination led to the induction of both local mucosal and systemic immune responses. This confirms the efficiency of VLPs to induce local immune responses and also validates the notion that systemic immune responses can be triggered through mucosal vaccination. Buonaguro's team is currently optimizing their VLP-based anti-HIV-1 vaccine (subtype A clade from Uganda) by means of a signal sequence of the transmembrane gp41 protein to obtain a trimeric form of the envelope [38]. Cells from HIV + and HIV- individuals treated *in vitro *with HIV VLPs did not show differences in the expression of cell surface markers such as CD83, HLA, CD80 or CD14. However, a difference in CD86 expression levels was observed between the two groups, suggesting that immune cells from seropositive individuals are capable of responding to the HIV antigens incorporated in the VLPs. The study of the pattern of cytokine production showed that IL-10 and IL-6 were more expressed in seropositive individuals than in seronegative controls. In the presence of VLPs, low doses of IL-10 were measured, suggesting that VLPs support a Th2 or a T regulatory pathway rather than a switch to Th1 response. Finally, the activation of both lymphokine clusters and IFN- stimulated gene clusters was confirmed in the PBMCs of infected individuals.

### Therapeutic vaccines

Julianna Lisziewicz and Esther Natz from Genetic Immunity in Budapest described the DermaVir Patch, a novel therapeutic vaccine against HIV/AIDS. The DermaVir Patch is their lead therapeutic vaccine candidate and originates from a development pipeline for plasmid- based vaccines [39]. The aim of this vaccine is to lower the viral load in HIV positive individuals by inducing immune responses of broad specificity against the virus. Key difficulties with plasmid-based vaccines, such as choice of construct, mode of delivery and formulation, have been specifically addressed during the development of this candidate. Hence the DermaVir Patch contains the full (mutated) viral genome providing coverage of the different HIV clades. The epitopes are combined in a single plasmid for maximal synergy, while reducing the total plasmid amount. Furthermore, the vaccine takes advantage of the properties of its carrier substance, mannose-polyethyleneimid (PEIm). Both the carrier and the vaccine formulation have been optimized to maximize plasmid uptake and release in DCs. This leads to efficient epitope presentation and long-lasting as well as specific immune responses, as demonstrated in immunogenicity and reduction of viral load in SIV-infected monkeys. Further hallmarks of this vaccine candidate is the delivery method (DermaPrep) on large areas of abraded skin to target Langerhans cells. A phase I clinical trial has demonstrated the safety of the vaccine and the DermaPrep delivery method. The vaccine is currently being tested in several placebo controlled phase II trials for immunogenicity and preliminary efficacy in treatment-naïve patients and patients receiving HAART.

## Microbicides

The emerging field of microbicide research is evolving rapidly, with several new innovative approaches, such as colorectal explants, freeze-dried tablets, new potential inhibitory molecules, and a vaccine-microbicide combination for mucosal immunisation. Carolina Herrera from St. George's Hospital, University of London, explored the use of drug combinations for colorectal application. To this end, the antiviral efficacy of two nucleoside reverse transcriptase inhibitors (NRTI) and two non- NRTIs (NNRTI), alone or in combination, were assessed in a colorectal explant model. The results clearly depicted the higher inhibitory activity of drug combinations compared to each drug alone. Furthermore, triple and quadruple combinations showed higher inhibitory activity than two drugs even against RTI escape mutants [40].

Freeze-dried muco-adhesive tablets, designed to overcome problems of poor mucosal retention and maintained gel structure, were presented by Manish Umrethia from Queen's University in Belfast. Carbopol, dapivirine and other polymer components were mixed to form multiple polymeric gels, and freeze-dried. *In vitro *testing of the gels and their freeze-dried variants demonstrated an advantage of the freeze-dried tablets. The latter displayed better stability, in addition to the higher viscosity and muco-adhesive properties compared to gels. Furthermore, there was no significant difference between the two formulations in the release of the antiviral compound dapivirine. In summary, due to their physicochemical properties, the freeze-dried tablets offer a prolonged vaginal residence time and a sustained release of antiviral compounds.

New inhibitory molecules based on natural substances in body fluids or sulfonamides were identified by Edward Karamov, Sylvaine Blois and William Paxton. Olipiphat™, a humic substance that is only moderately toxic, had a pronounced dose-dependent activity towards both AZT-sensitive and resistant HIV strains *in vitro*. In addition, Olipiphat™ showed synergistic effects with the nucleoside RT inhibitor AZT. Paxton reported two molecules that can block HIV-1 capture and transfer through binding to DC-SIGN expressed on DCs. One molecule, bile-salt stimulated lipase (BSSL), was isolated from human milk and the other, mucin6 (MUC6), from the seminal plasma. They have similarities in structure and in their specific glycosylation patterns, which likely facilitates their binding efficiencies [41].

### Antibodies as microbicides

Andrea Gorlani (PhD student) from Utrecht University emphasised the importance of microbicide development and introduced the use of llama heavy-chain antibody fragments (VHH) combined with topical microbicides. Immunoglobulin of the *Camelidae *family, devoid of the light chains [42], have been reported to show neutralising properties and high affinity for HIV-1 gp120 [43]. Gorlani presented results showing how these VHH can fulfill the criteria for use in a successful HIV microbicide. The requirements for a microbicide include stability, effective formulation, tissue permeability and low cost. The VHH were produced in a fed-batch fermentation system, followed by purification, a method that can easily be scaled up at low cost. The VHH have been formulated in a vaginal gel as well as in novel intravaginal ring devices, and shown to be stable in both. Stability was sustained in harsh conditions such as high temperature and low pH. Permeability through vaginal mucosa was demonstrated and showed a satisfactory rate through both intact and damaged epithelium. In summary, Gorlani showed that llama heavy-chain antibody fragments binding HIV gp120 can be used as entry inhibitors and applied as topical microbicides.

A series of posters addressed various hurdles in microbicide research. Since microbicides will inevitably be used also by undiagnosed HIV + women, there is growing concern about acquiring resistance to HIV if antiviral agents are incorporated in the microbicide. Katrijn Grupping (PhD student) showed that high level resistance to two microbicide candidate CD4 binding site (bs) inhibitors was easily induced *in vitro*, requiring only a few amino acid changes while displaying cross-resistance with other CD4 bs inhibitors. However, this should not be a problem as these inhibitors are not used in therapy. Reverse transcriptase inhibitors are, however, essential in HIV-1 therapy and their use as microbicides could narrow systemic therapeutic options, as demonstrated by Philippe Selhorst (PhD student). Thus, RTI microbicides might promote the selective transmission of resistant virus [44,45]. Therefore, as in systemic treatment, the solution seems to be a combination of different drug classes. In this context there is promising news as Sylvain Blois from the University of Cagliari has discovered a new class of HIV-1 inhibitors which seem to be active against the conserved nucleocapsid protein 7, resulting in production of defective virus. While these benzene sulfonamides are active only at micromolar levels, they compensate with their broad spectrum activity and structural simplicity.

The next important issue is microbicide delivery. Youssef Gali et al. (PhD student, Institute of Tropical Medicine) have measured the toxicity profile *in vitro *of different pharmaceutical excipients in vaginal microbicide formulations. Their study revealed that excipients show a distinct hierarchy in their potential to exert toxic effects and that this should be addressed when considering their inclusion in developing new formulations. One novel approach to optimize mucosal protection would be to combine microbicides with a mucosal vaccine. To this end, Katja Klein (PhD student) screened different permeation enhancers as potential antigen carriers for mucosal delivery. She demonstrated that at least some of the compounds were able to increase the bioavailability of vaccine antigens through the vaginal route. Donatella Negri et al. (Istituto Superiore di Sanità, Rome), demonstrated that sublingual immunisation also showed promise as an alternative route for vaccine delivery as it induces a persistent immune response in mice. Umrethia et al. focused on optimizing the administration of mucosal vaccines or microbicides containing gp41 constructs. Freeze-dried formulations were developed which are suitable for administration via sublingual and vaginal routes. The lyophilized formats can release gp41 molecules at a high rate, have an increased antigen stability and are easy to apply, representing a useful tool for the development of microbicides.

### Novel adjuvant approaches

Two approaches for HIV vaccination have been investigated. One strategy is based on the activation of DC by apoptotic cells [46]. Apoptotic cells obtained from γ-irradiated DCs were used as an adjuvant for DNA vaccination in a proof-of-concept study ([47] and unpublished data). Macaques were immunised with autologous apoptotic activated cells that had previously been infected *ex vivo *with replication defective SIVmac239ΔEnv/VSVEnv pseudovirus. Activated but not resting apoptotic cells proved to be adequate adjuvants for systemic IgG and mucosal IgA production. Three intradermal immunisations induced IFN-γ production, Th1 and CD8 + T-cell responses as well as neutralising antibodies and no detectable levels of virus replication. The second strategy involves biocompatible microspheres (H1D) as a delivery system for DNA and protein vaccines.

Microspheres have been hypothesised to favour the uptake of protein, induce maturation of APC, protect and permit a controlled release of antigen. Cynomolgus monkeys immunised with biologically active HIV-1 Tat protein adsorbed on H1D microspheres showed a significant control of viremia after challenge which correlated with the preservation of CD4 + T-cells. One hypothesis is that vaccine modalities that specifically improve T-helper cell responses might lead to better protection. Sieghart Sopper (German Primate Center, Göttingen) showed in a macaque/SIV model for AIDS that expression of activation markers are related to higher viral load and disease progression as early as four weeks after infection. Vaccination using different prime boost regimens, which reduced acute and post-acute viral load, resulted in earlier activation of CD4 + T-cells [48]. These results suggest that T-helper cells may contribute to the containment of viral replication during acute infection in macaques.

## Conclusion

The conference presentations focused on common goals of developing effective HIV prevention strategies. EUROPRISE brings together scientists from both microbicide and vaccine fields. The program focuses on the premise that vaccines and microbicides that target multiple stages of viral transmission through the mucosa will have the best chances of success. To demonstrate such approaches several partners are involved in clinical trials. The meeting in Budapest was focused on collaborative work between partners and was largely presented by the shared PhD students of the network. The showcasing of presentations by PhD students at the meeting promises a bright future for HIV research within Europe.

## Competing interests

The authors declare that they have no competing interests.

## Authors' contributions

All authors participated at the EUROPRISE conference as to be able to report on it. MB, AC, KDC, WDH, TD, SD, KG, DH, JH, KK, LM, PP, MR, JS, PS, AS, MJVG, and CW were in charge of the writing of dedicated chapters covering the different sessions of the conference. GS, BW and RS organized the sessions and the writing. Together with PB they wrote, corrected and revised the manuscript. All authors read and approved the final manuscript.
