# Correction: Rational design of HIV vaccine and microbicides: report of the EUROPRISE annual conference

**DOI:** 10.1186/1479-5876-8-82

**Published:** 2010-09-03

**Authors:** Britta Wahren, Priscilla Biswas, Marie Borggren, Adam Coleman, Kelly Da Costa, Winni De Haes, Tessa Dieltjens, Stefania Dispinseri, Katrijn Grupping, David Hallengärd, Julia Hornig, Katja Klein, Lara Mainetti, Paolo Palma, Marc Reudelsterz, Janna Seifried, Philippe Selhorst, Annette Sköld, Hannes Uchtenhagen, Marit J van Gils, Caroline Weber, Robin Shattock, Gabriella Scarlatti

**Affiliations:** 1 Karolinska Institute, Stockholm, Sweden; 2 San Raffaele Scientific Institute, Milan, Italy; 3 Lund University, Lund, Sweden; 4 Imperial College, London, UK; 5 St. George University, London, UK; 6 Institute of Tropical Medicine, Antwerp, Belgium; 7 Università degli Studi di Milano, Milan, Italy; 8 Università Vita-Salute San Raffaele, Milan, Italy; 9 University of Rome "Tor Vergata", Ospedale Pediatrico Bambino Gesù, Rome, Italy; 10 Robert Koch Institute, Berlin, Germany; 11 Institut de Biologie et Chimie des Protéines, Lyon, France; 12 Academic Medical Center, Amsterdam, the Netherlands

## Correction

Following the publication of this article [1], it was noted that a co-author had been omitted from the authors list. The submitting authors would like to apologise to Hannes Uchtenhagen for this error. The Competing interests and Authors' contributions sections have now been updated to reflect this amendment.

## Competing interests

The authors declare that they have no competing interests.

## Authors' contributions

All authors participated at the EUROPRISE conference as to be able to report on it. MB, AC, KDC, WDH, TD, SD, KG, DH, JH, KK, LM, PP, MR, JS, PS, AS, HU, MJVG, and CW were in charge of the writing of dedicated chapters covering the different sessions of the conference. GS, BW and RS organized the sessions and the writing. Together with PB they wrote, corrected and revised the manuscript. All authors read and approved the final manuscript.
